# Long-term prognosis of unheralded myocardial infarction vs chronic angina; role of sex and coronary atherosclerosis burden

**DOI:** 10.1186/s12872-018-0890-5

**Published:** 2018-07-31

**Authors:** Clara Carpeggiani, Claudio Michelassi, Patrizia Landi, Antonio L’Abbate

**Affiliations:** 1 CNR Institute of Clinical Physiology, Via Moruzzi, 1, 56124 Pisa, Italy; 2 Scuola Superiore Sant’Anna, Institute of Life Sciences, Piazza Martiri della Libertà 33, Pisa, Italy

**Keywords:** Angina pectoris, Myocardial infarction, Prognosis, Coronary atherosclerosis, Sex

## Abstract

**Background:**

Angina pectoris (AP) and unheralded myocardial infarction (MI) are considered random clinical equivalents of ischemic heart disease (IHD). Aim of the study was to evaluate the long-term progression of AP as opposed to unheralded MI as alternative first clinical presentations of IHD and the effect of sex on prognosis.

**Methods:**

The study included 2272 consecutive patients, 1419 MI and 1353 AP, hospitalized from 1995 to 2007 at CNR Clinical Physiology Institute, Pisa, Italy and followed up to December 2013, who fulfilled the following criteria: unheralded MI or AP as first manifestation of IHD; age < = 70 years; known coronary anatomy; at least 10-year follow-up. Fatal and non fatal MI, all-cause, and cardiac deaths were the end-points.

**Results:**

Males were predominant in MI (86%) as compared to AP (77%). Females were predominantly affected by AP (61%, MI 39%), and older than men (61 ± 7 vs 59 ± 8 years, *p* < 0.001). Coronary stenoses were prevalent in MI. During 115 ± 58 months follow-up, 628 deaths (23%) were observed, including 269 cardiac (43%), and 149 cancer deaths (24%). Long-term prognosis was significantly better in AP than MI group. The lowest prevalence of future MI was recorded in female AP (*p* < 0.001).

**Conclusions:**

MI as first clinical manifestation of IHD implies a more adverse prognosis than AP; future MI is a rare event in AP; sex influences the first presentation of IHD and its course with possible implications for preventive strategy.

**Electronic supplementary material:**

The online version of this article (10.1186/s12872-018-0890-5) contains supplementary material, which is available to authorized users.

## Background

It is commonly noted that the clinical manifestations of ischemic heart disease (IHD) vary widely, starting from its initial presentation to its progression over time. This is the case of acute unheralded myocardial infarction (MI) as first manifestation compared to episodes of angina pectoris (AP), which may persist for years without progressing to MI. Although generally considered random clinical equivalents of the same pathological process, i.e., coronary artery disease [1, 2], the occurrence of these two main clinical features and their sequence can vary in the same patients as well as in different patients [3–5]. Fatal and non-fatal MI as first clinical manifestation of IHD has been reported to be lower than the manifestation of angina [6]**,** which is considered to be the most prevalent manifestation of IHD, affecting up to 5% of the population aged over 40 years in most developed countries [7, 8]**,** favoring female sex [6, 9]**.** On the other hand, it is a common opinion that the two clinical manifestations are frequently consequential, AP being considered to have a ‘natural tendency’ to evolve toward MI, so that early identification and treatment of AP is regarded as an important goal to avert the progression to MI [10]. However, contemporary information on their long-term prognosis and how frequently AP evolves to MI is relatively sparse and not clearly defined [11]**.**

Aim of the study was to compare the long-term follow-up (at least 10 years) of a cohort of consecutive patients with documented unheralded MI or chronic AP as initial presentation of the disease to estimate the putative differences in long-term lifetime risk of each distinctive phenotype and to evaluate the influence of cardiovascular risk factors, with particular emphasis on sex differences in MI occurrence as first presentation and long-term risk. In recent years an increased inappropriateness in the diagnosis and therapy of IHD patients has been noted irrespective of the clinical IHD phenotype, with health and economic implications [12]. Better knowledge of the long-term course of different clinical presentations of IHD might improve appropriateness of diagnostic and therapeutic strategies, reducing health costs.

## Methods

### Patient selection

The study included 2772 consecutive patients admitted to the CNR Institute of Clinical Physiology, Pisa, Italy due to IHD over a 12-year period (from 1995 to 2007), who fulfilled the following criteria: sudden unheralded MI without history of previous AP (group MI), patients with chronic AP (group AP), without history of previous MI or congestive heart failure; documentation of transient myocardial ischemia; age < = 70; submitted to coronary angiography at the time of hospitalization; available follow-up information. At discharge, all demographic, history, clinical and instrumental data were collected in the Institute’s dedicated cardiovascular database- IMAGE [13]. For this study, data on the ‘onset type’ of IHD (which could precede hospitalization in the Institute and thus enrollment in the present study, even years before), and its progression features, risk factors, clinical features at the time of hospitalization and diagnosis. The definition of chronic AP was based on the ACC/AHA clinical statement and documentation of inducible ischemia on ECG and/or imaging stress test. Traditional risk factors were defined for each patient: basal obesity: BMI > 30; diabetes mellitus: fasting plasma glucose > 126 mg/dl out of treatment; arterial hypertension: systolic blood pressure > 140 mmHg and/or diastolic pressure > 90 mmHg out of treatment; hypercholesterolemia: fasting plasma total cholesterol > 200 mg/dl out of treatment, hypertriglyceridemia: fasting plasma triglycerides > 160 mg /dl out of treatment. Coronary artery disease (CAD) was defined by the number of major coronary vessels affected by > 50% lumen reduction (left main coronary branch being considered equivalent to two vessels) at invasive coronary angiography. Patients with normal coronary vessels were included when diagnosis of angina and ischemia at rest was present at discharge, and /or vasospastic angina was suspected and /or documented. Patients presenting with unstable angina as well as patients with both MI and angina were excluded. Moreover patients who had congenital heart disease, hypertrophic cardiomyopathy, or significant valvular heart disease other than mitral insufficiency thought to be secondary to IHD were discarded.

A total of 1419 patients were selected in group MI (1101 STEMI, 318 NSTEMI) and 1353 in group AP with an even distribution (51 and 49% respectively).

### Follow-up

For each patient, follow-up began at discharge and was planned for a minimum period of 10 years. For the purposes of the present study it was concluded in December 2013. Follow-up data were obtained in at least one of the following ways: from the patient’s hospital record, by contacting the patient’s physician, and by telephone interview conducted by trained personnel, during periodic scheduled visits at the outpatient clinic. The only endpoints considered were major events such as non-fatal MI and death classified as cardiac death and non-cardiac death. The diagnosis of MI during the follow-up period required a hospital discharge chart documenting and defining, when possible, the acute event (Q or non-Q MI and site). Medical records or death certificates were used to obtain cause of death. The documentation of life-threatening arrhythmias, myocardial infarction or cardiac arrest, or death attributable to congestive heart failure was required to make diagnosis of cardiac death, in the absence of any other precipitating factor. Coronary revascularization procedures during the follow-up were also reported.

### Statistical analysis

Student’s t-test or analysis of variance as appropriate was used to test for significant difference in quantitative measures and the χ ^2^ test for the qualitative parameters. The Cox proportional hazard regression model was used to explore the possible independent association between the two phenotypes of IHD and gender and the major endpoints such as non-fatal MI and death. The regression model included known potential confounders such as age and traditional cardiovascular risk factors. Hazard ratios (HR) with 95% Confidence interval (CI) were calculated on the entire study population. Kaplan Meyer survival curves were analyzed for the two groups and for the two genders. Analyses were performed with the statistical package SPSS (version 20).

## Results

**Fig. 1:**
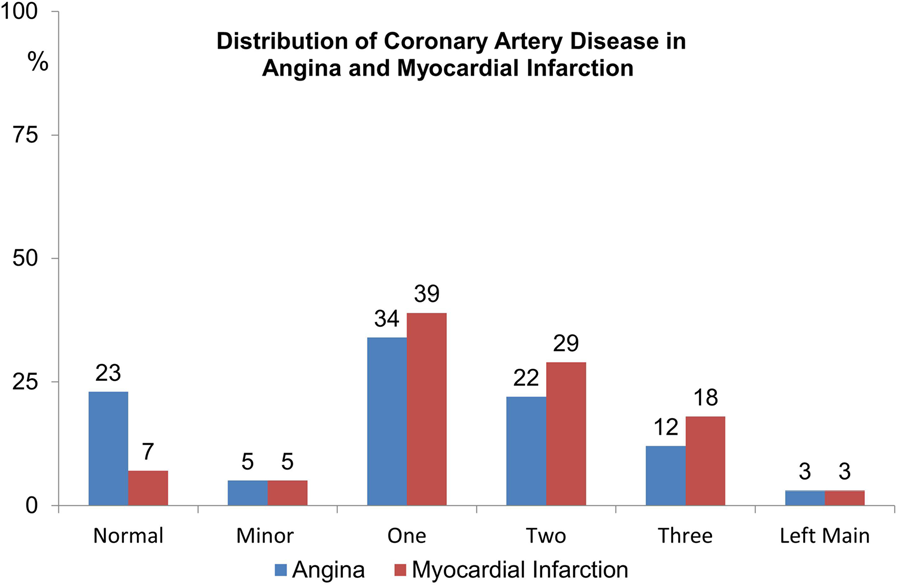
Distribution of Coronary Artery Disease in Angina and Myocardial Infarction. The number of diseased coronary vessels (main vessels with > 50% lumen reduction) is reported for angina pectoris and myocardial infarction groups. Normal = normal vessels; minor: minor vessels

The characteristics of the entire population of 2772 patients, at study entry and stratified according to IHD initial presentation and by gender, are shown in Table 1 (see also Additional file 1: Table S1A). Males were largely prevalent in both the MI (86%) and the AP groups (77%). The two groups differed regarding age at the onset of IHD and at hospitalization, the youngest being MI males and the oldest AP females. AP patients more often had a history of hypertension and higher levels of total cholesterol, LDL and HDL at enrollment. MI patients were more frequently smokers (66% vs 49%) with slightly higher level of glycaemia at hospital entry. Severity of angiographic coronary atherosclerosis (mean number of main coronary vessels with > 50% lumen reduction) prevailed in MI patients. The distribution of the diseased coronary vessels is shown in Fig. 1. Previous to enrollment, 530 patients had a coronary revascularization procedure that was prevalent in the MI group (Table 1).

**Table 1:** Clinical characteristics, medical treatment and coronary revascularization procedures at enrollment in the entire population stratified by the first clinical presentation of IHD (Angina vs unheralded Myocardial infarction)

	Total 2772	Angina (1353)	Myocardial Infarction (1419)	*p*-value
Sex males, N(%)	2250 (81)	1033 (76)	1217 (86)	*0.001*
Age at disease onset (yrs)	55 ± 9	56 ± 9	54 ± 9	*< 0.001*
Age at hospitalization (yrs)	59 ± 8	60 ± 7	58 ± 8	*< 0.001*
IHD Family history, N(%)	1411 (51)	716 (53)	695 (49)	0.038
Smoking, N(%)	1588 (57)	656 (49)	932 (66)	*< 0.001*
Obesity, N(%)	795 (29)	400 (30)	395 (28)	*315*
BMI, mean ± SD	27.5	28 ± 4	27 ± 4	*0.363*
Diabetes, N(%)	576 (21)	273 (20)	303 (21)	0.446
Hypertension, N(%)	1439 (52)	779 (58)	660 (47)	*0.001*
Hypercholesterolemia, N(%)	1923 (69)	954 (71)	969 (68)	0.204
Hypertriglyceridemia, N(%)	735 (27)	365 (27)	370 (26)	0.591
Glycaemia, mean ± SD	111 ± 49	107 ± 38	115 ± 57	*< 0.001*
Systolic Blood Pressure	133 ± 21	136 ± 20	130 ± 22	*< 0.001*
Diastolic Blood Pressure	76 ± 11	76 ± 11	75 ± 12	*< 0.001*
Total Cholesterol mean ± SD	198 ± 46	204 ± 45	193 ± 47	*< 0.001*
HDL	41 ± 15	43 ± 15	39 ± 16	*< 0.001*
LDL	123 ± 45	129 ± 49	118 ± 40	*< 0.001*
Triglycerides	146 ± 97	147 ± 96	146 ± 98	*0.825*
Previous CABG, N(%)	194 (7)	61 (5)	133 (9)	*< 0.001*
Previous PCI, N(%)	382 (14)	146 (11)	236 (17)	*< 0.001*
Coronary Atherosclerosis	1.47 ± 1.03	1.31 ± 1.05	1.64 ± 0.94	*< 0.001*
EF%	53 ± 10	57 ± 7	49 ± 11	*< 0.001*
*Medications (%)*				
Anti-platelet agents	2371 (86)	1108 (82)	1263 (89)	*< 0.001*
Calcium channel blockers	832 (30)	502 (37)	330 (23)	*< 0.001*
Nitrates	1792 (65)	830 (61)	962 (68)	*< 0.001*
ACE Inhibitors	943 (34)	332 (25)	611 (43)	*< 0.001*
Betablockers	1320 (48)	543 (40)	777 (55)	*< 0.001*
Statins	1514 (55)	706 (52)	808 (57)	0.011
Antidiabetics	429 (16)	202 (15)	227 (16)	0.437

*IHD* Ischemic Heart Disease, *BMI* Body Mass Index, *PCI* Percutaneous Coronary Intervention, *CABG* Coronary Artery Bypass Graft, Coronary Atherosclerosis (number of main coronary vessels, with > 50% lumen reduction, mean ± SD); *EF* Ejection Fraction

Stratification of the two groups by gender (Additional file 1: Table S1A) showed that compared to males, females were predominantly affected by AP (61% vs 39% MI) and were significantly older (61 ± 7 vs 59 ± 8 years, *p* < 0.001), although the difference in age with regard to the prevalence of MI and AP was significant only in men (AP 57 ± 9 vs MI 57 ± 9 years in females *p* = 0.79, AP 56 ± 8 vs MI 53 ± 9 years in men, *p* < 0.001). Independently from gender stratification, MI patients confirmed the prevalence of coronary stenosis, and AP patients confirmed the prevalence of hypercholesterolemia and arterial hypertension.

Of the 1419 MI, 1105 (78%) were Q and 314 (22%) non-Q. Regarding the site of necrosis, 668 (47%) were inferior, 505 (36%) anterior, and 246 (17%) lateral. The inferior site was more frequent in males compared to females (49% vs 34%), while the anterior (43% vs 34%) and lateral (23% vs 16%) sites were more frequent among females (*p* < 0.001 between genders in all cases). The fraction of non-Q relative to Q infarction was higher in females than in males (31% vs 21% *p* = 0.02).

### Follow-up

During the average follow-up period of 115 ± 58 months, the clinical course was more favorable in Group AP. A total of 628 deaths (23%), including 269 (43%) cardiac deaths, 239 new non-fatal MI were recorded. The distribution of outcome events and the type of death in the two groups is reported in Table 2. The rate of coronary revascularizations was significantly higher in the MI group (1063, 76% vs 892, 65% respectively, *p* < 0.001).

**Table 2:** Aggregate Outcomes in the study population subdivided according to the IHD first clinical presentation (Angina vs unheralded MI)

	Angina (1353)	Myocardial Infarction (1419)	*P*
*Follow-up (months)*	120 ± 77	132 ± 199	0.086
Death, N (%)	227 (17)	401 (28)	< 0.001
Cardiac, N (%)	74 (6)	195 (14)	*< 0.001*
Non-Fatal Myocardial infarction, N (%)	97 (7)	142 (10)	0.008
Cancer, N (%)	161 (12)	158 (11)	0.552
CR N, (%)	892 (66)	1063 (75)	< 0.001
*Cause of death*			
*Cardiac*	74	195	
Myocardial infarction	28	79	
Sudden Death	8	23	
Heart failure	13	40	
Cardiac Surgery	5	6	
Other cardiac cause	20	47	
*Non-cardiac*	153	206	
Vascular	11	22	
Cancer	67	82	
Other Surgery	1	2	
Renal or Hepatic failure	2	7	
Accident	4	2	
Infectious	4	3	
*Other causes*	64	88	

*IHD* ischemic heart disease, *CR* coronary revascularization

#### New MI

**Fig. 2:**
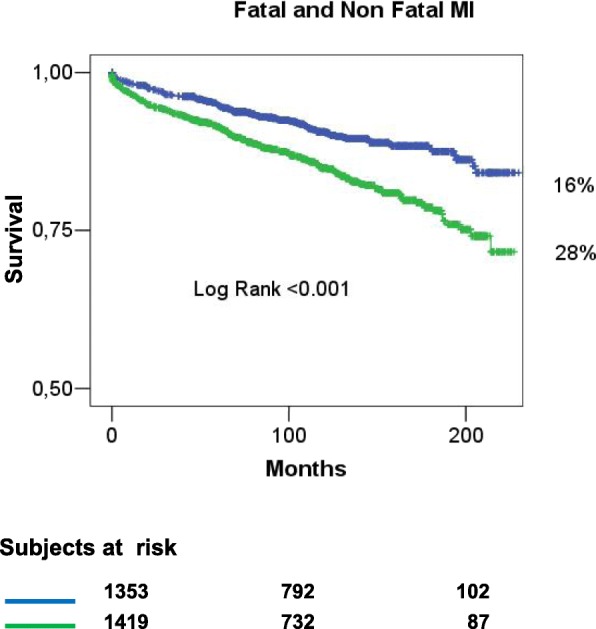
Kaplan Meyer curves for fatal and non-fatal myocardial infarction in angina pectoris (blue line) and myocardial infarction (green line). *MI* myocardial infarction

**Fig. 3:**
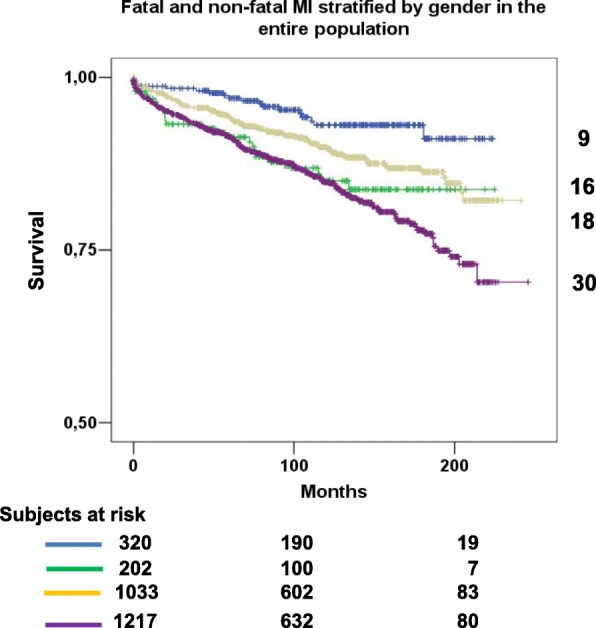
Kaplan Meyer curves for fatal and non-fatal myocardial infarction in angina pectoris and myocardial infarction stratified by gender. MI = myocardial infarction. Blue line: females in angina group; yellow line: males in angina group; green line: females in myocardial infarction group; purple line: males in myocardial infarction group

A new, fatal or non-fatal MI was prevalent in the MI group (Table 2). The Kaplan-Meier estimates of MI occurrence at approximately 20 years were 28 and 16% respectively for MI (fatal and non-fatal) and AP group (*p* < 0.001) (Fig. 2). In the AP group the occurrence of MI in the follow-up was still significantly lower in females than in males (9% vs 18% respectively, *p* = 0.015), while no significant difference was found between sex in the MI group (16% in females vs 30% in males, *p* = 0.453) (Fig. 3).

The Kaplan-Meier estimates of MI occurrence was also performed excluding patients with normal coronary vessels. Very similar results were obtained and the Kaplan Meyer estimates of MI (fatal and non-fatal) were 29 and 20% respectively in the MI and AP group (*p* < 0.001). The new gender stratification analysis is reported in Additional file 2: Figure S3a.

In the majority of patients (71% of AP, 68% of MI) new MI occurred months or even years after coronary revascularization (81 ± 46 months in AP, 79 ± 51 in MI) and in four patients of the AP group new MI occurred as a complication of the revascularization procedure.

The site of the new MI was known in only 103 patients, 60 of MI and 43 of AP group: 43 were anterior, 43 inferior and 17 lateral.

Additional file 3 Table S3 shows the risk of a new MI in the follow-up associated with baseline clinical characteristics. The multivariable Cox analysis confirmed that a new MI was prevalent in males with previous MI and evidence of wider extent of coronary atherosclerosis.

#### Death

History of MI was also predictive of death, both cardiac and non-cardiac (Additional file 3: Table S3). The most adverse prognosis was observed in patients with metabolic disorders such as diabetes, especially in those without coronary revascularization. The Kaplan-Meier estimates of cardiac and all-cause death at almost 20 years was significantly different between MI and AP groups (*p* < 0.0001) (Additional file 4 Figure S4 and Additional file 5: Figure S5), without any significant difference between genders.

## Discussion

The main results of the study were the following: 1. Long-term survival in patients with IHD was markedly poorer in those with MI as first clinical manifestation of the disease as compared to AP; 2. Occurrence of MI during the follow-up was more frequent in patients who had already a MI than in the others. 3. Coronary atherosclerosis was prevalent in MI independently by sex. 4. Female sex was preferentially associated with AP course, had a significantly lower incidence of MI during the follow-up and a better long-term outcome.

To our knowledge, this is the first report that correlates the long-term prognosis of IHD to the modality of its first clinical manifestation and underlines that patients with persistent episodes of angina but free of MI infrequently report MI during the follow-up, differently from patients with initial MI. Moreover, our study underlines the role of sex in the clinical phenotype of IHD and its progression.

### Comparison with previous studies

Our study expands the investigation to a relatively large cohort of well characterized patients with IHD with a mean follow-up period of 10 years, and in particular provides additional evidence regarding the follow-up events in the two IHD phenotypes which did never overlap until the index hospitalization and database inclusion. It is important to consider, that the overlap between the two phenotypes is quite common in the populations enrolled in many studies which makes quite difficult to compare the results [14–16].

In the present study the proportion of MI and AP patients was quite similar, which is in contrast with the reported lower prevalence of MI as first presentation of IHD as compared to angina without infarction [6]. A recent report from American Heart Association reports a prevalence around 2.8% for MI and 3.8 for AP [17]. The discrepancy with our population could be due to different selection criteria. Patients in our study were consecutive and < = 70 years old, most admitted *for* special investigation and treatment as coronary angiography and or PCI, without history of congestive heart failure (group AP). Another reason could be the different prevalence of AP and MI in different countries. In the Hemingway meta-analysis, angina prevalence varied approximately 20-fold between countries, ranging from 0.73 to 14.4% in women and 0.76 to 15.1% in men [18]. In Italy the reported prevalence of the two phenotypes is very similar and this could explain our results [19, 20].

The overall prognosis was significantly more benign in AP and in particular the incidence of new MI during follow-up (0.9/100 patient years vs 1.6/100 patient years, *p* < 0.001), which was lower than the one reported by Daly et al. (3.2/100 patient years, CI 2.3–4.4) [21] and in other trials (ranging from 1.1 to 1.5%) [21–24]. The re-infarction rate was also in the lower range of the one reported in the literature (range 1.8–6.9% year in different studies, 95% CI 0.97–1.77, *P* = 0.08.) [25]. The difference might reflect the duration of follow-up, age, inclusion and exclusion criteria, coronary atherosclerosis involvement, type of treatment. This study included in both groups, with a prevalence in AP (25% vs 7%), patients with normal coronary vessels who have a well known good prognosis. However, the Kaplan-Meier estimates of MI occurrence performed excluding patients with normal coronary vessels obtained similar results (Additional file 2: Figure S3a). Our study was a single-center study and all patients received similar treatment at discharge as secondary prevention and revascularization procedure when required.

In the literature, MI databases report on the significance of angina before and/or after MI [3, 5, 16, 26], and stress the association of angina with higher likelihood of readmission to the hospital, and higher risk of events [26] but can hardly be compared with our results due to different criteria of patients’ selection, modalities and duration of follow-up.

The severity and location of the coronary lesions are among the main factors that have been compared in the two ischemic syndromes in the literature and that have been taken into account to explain the differences in clinical outcomes [27, 28]. A number of studies reported that patients with AP have more severe and extensive coronary lesions than patients with acute unheralded MI [29]. According with more recent papers [16] we found a higher coronary atherosclerotic involvement (expressed as average number of main coronary diseased vessels at target coronary angiography) in MI compared to AP patients, and the extent of coronary atherosclerosis was predictive of new infarct during the follow-up.

### IHD and sex

A major result of the study was that the different association of sex with the two manifestations of IHD does not wane over time. In contrast with previous studies [6, 18, 30, 31], we compared the two IHD phenotypes of similar age and followed them for a longer time period to investigate the persisting long term effect of sex on MI occurrence independently by age. In the AP group, the incidence of new MI was still significantly lower in females, while no significant difference between sexes was found in the MI group (Fig. 3). On the contrary, sex did not have any significant effect on cardiac and non-cardiac death at approximately 20 years (Additional file 4: Figure S4 and Additional file 5: Figure S5). To the best of our knowledge, this long-term different effect of sex on the two phenotypes of IHD and on (re)infarction rate has never been reported in a large population.

The mechanisms through which sex participates in the pathogenesis of IHD remains speculative [32]. It is assumed that exposure to endogenous estrogens during the fertile period of life delays the development of atherosclerosis in women. In the Women’s Ischemia Syndrome Evaluation (WISE) study it was shown that young women with endogenous estrogen deficiency have a more than sevenfold increase in coronary event risk [33]. Our results showed that the protective effect in females was evident in the AP group and persisted after menopausal age, preventing acute cardiac instability and MI. On the other hand the female sex was not protective against cardiac and total mortality in both IHD phenotypes (MI or AP). This was in accord with previous report on the adverse IHD prognosis in women [33]. Adverse coronary reactivity and microvascular dysfunction have been suggested as contributory factors to a female-specific myocardial ischemia pathophysiology [32]. Moreover differences in medical strategy in the follow-up comparing to men cannot be excluded. Finally, new MI in females could have been underestimated particularly if considered that sex did not have any significant effect on cardiac and non-cardiac death. It’s known that women compared to men are more frequently underdiagnosed for acute MI and they are less frequently studied with coronary angiography.

### Other risk factors

Regarding the other conventional risk factors no consistent pattern has emerged from this study as well as from the literature [34–37]. AP patients had a history of hypertension more often than did MI patients as well as higher levels of total cholesterol and LDL at entry. Differently, MI patients were prevalently smokers (66% vs 49%) with slightly higher levels of glycaemia at enrollment.

### Study limitations

Our study has several potential limitations. It was a single-center study, which, on the other way, meant similar treatment at discharge.

The type and number of events occurring in the two groups from disease onset to the time of enrollment in the study were carefully taken into account. However, it should be considered that the patients enrolled in the study were only those newly affected by IHD who survived from the onset of the disease up to the time of enrollment. In other words, the incidence of death in our study as well as the final disparity in outcome between the two groups were underestimated in proportion to the time elapsed between onset and enrollment (average 4 years in our study).

During the follow-up we had no information either on the site of the new infarct in all patients or on the potential progression of coronary disease in the follow-up in those not undergoing a new coronary angiography. As such, we cannot definitively draw conclusions on the coronary atherosclerotic burden leading to new MI.

We did not use time-dependent covariates in the Cox model and we cannot exclude the effect of changes in life style, such as smoking cessation on mortality curves.

Moreover, we did not have detailed information on the interim changes in medical treatment as compared to time of enrollment. At discharge from the hospital, medical treatment and lifestyle recommendations did not differ substantially in the two groups and were generally comparable in women and men. During the follow-up medical therapy was left to the clinical judgment of the referring physician-cardiologist. Some chronic medical therapy are associated to a reduced risk of events, such as aspirin or statins or beta-blockers and angiotensin-converting enzyme inhibitors. Although we did not have detailed information regarding medical treatment during follow-up, we feel confident that patients adhered to the best therapy according to physician recommendations.

## Conclusion

We found that the first clinical manifestation of the disease (i.e., unheralded MI or transient episodes of myocardial ischemia (angina pectoris) without necrosis) was per se predictive of the long-term outcome, the patients with initial MI having more coronary atherosclerosis burden and being more prone to re-infarction and late cardiac death. An unfavorable course of the disease was more frequently observed in males than in females. Female sex was preferentially associated with the anginal presentation of the disease, had less coronary atherosclerosis involvement and had lower incidence of MI during follow-up.

According to the above results we may speculate that the way of presentation of the IHD together with the sex may greatly help in predicting the long-term prognosis of the disease and tailoring the conservative vs aggressive secondary prevention strategy. A large proportion of female patients with IHD but free of initial MI will likely perform well with the adoption of a conservative strategy and medical treatment.

## Additional files

Additional file 1: **Table S1.** Clinical characteristics of study population stratified by first presentation of IHD and gender. (DOCX 21 kb)

Additional file 2: **Figure S3a.** Kaplan Meyer curves for fatal and non-fatal myocardial infarction in angina pectoris and myocardial infarction excluding patients with normal coronary vessels, stratified by gender. MI = myocardial infarction. Blue line: females in angina group; yellow line: males in angina group; green line: females in myocardial infarction group; purple line: males in myocardial infarction group. (PPTX 118 kb)

Additional file 3: **Table S3.** Cox regression estimates of the hazard ratios associated with major follow up events. (DOCX 18 kb)

Additional file 4: **Figure 4.** Kaplan Meyer survival curves for cardiac death in angina pectoris and myocardial infarction stratified by gender. Blue line: females in angina group; yellow line: males in angina group; green line: females in myocardial infarction group; purple line: males in myocardial infarction group. (PPT 135 kb)

Additional file 5: **Figure 5.** Kaplan Meyer survival curves for all-cause death in angina pectoris and myocardial infarction stratified by gender. Blue line: females in angina group; yellow line: males in angina group; green line: females in myocardial infarction group; purple line: males in myocardial infarction group. (PPTX 103 kb)

## Abbreviations

AP: Angina pectoris
BMI: Body mass index
CAD: Coronary artery disease
CI: Confidence interval
HR: Hazard ratio
IHD: Ischemic heart disease
MI: Myocardial infarction
