# Supplementary material for: Long-term prognosis of unheralded myocardial infarction vs chronic angina; role of sex and coronary atherosclerosis burden
Source: BMC Cardiovasc Disord. 2018 Jul 31;18:156. doi: 10.1186/s12872-018-0890-5 (PMC6069774; doi:10.1186/s12872-018-0890-5)
Supplement: Supplementary file 1 — Table S1. Clinical characteristics of study population stratified by first presentation of IHD and gender. (DOCX 21 kb) [file 12872_2018_890_MOESM1_ESM.docx]

| **Table IA**. **Clinical characteristics of study population stratified by first presentation of IHD and gender.** | | | | | | |  | |  | | |  | |  | |  | |  | |
| --- | --- | --- | --- | --- | --- | --- | --- | --- | --- | --- | --- | --- | --- | --- | --- | --- | --- | --- | --- |
|  | | | Females | | Males | | | | | |  | | | | |  | |  | |
|  | Angina  (320-23%) | Myocardial Infarction  (202-14%) | | P-value | | Angina  (1033-77%) | | Myocardial Infarction  (1217-86%) | | P-value | | |  | |  | |  | |  |
| Age at disease onset(yrs)  Age at hospitalization(yrs) | 57±9  61±6 | 57±9  60±8 | | 0.79  0.45 | | **56±8**  **59±8** | | **53±9**  **58±8** | | **<0.001**  **<0.001** | | |  | |  | |  | |  |
| IHD Family history (%) | 59 | 55 | | 0.32 | | 51 | | 48 | | 0.18 | | |  | |  | |  | |  |
| Smoking (%) | 29 | 46 | | 0.35 | | **54** | | **69** | | **<0.001** | | |  | |  | |  | |  |
| Obesity (%)  Body Mass Index | **39**  28±5 | **35**  27±5 | | **<0.001**  0.17 | | 27  28±4 | | 27  28±4 | | 1.00  0.66 | | |  | |  | |  | |  |
| Diabetes (%) | 22 | 25 | | 0.34 | | 20 | | 21 | | 0.59 | | |  | |  | |  | |  |
| Hypertension (%) | **61** | **51** | | **0.03** | | **57** | | **40** | | **<0.001** | | |  | |  | |  | |  |
| Hypercholesterolemia (%) | 73 | 69 | | 0.37 | | 70 | | 68 | | 0.44 | | |  | |  | |  | |  |
| Hypertriglyceridemia (%)  Glycaemia  Systolic Blood Pressure  Diastolic Blood Pressure  Total Cholesterol  HDL  LDL  Triglycerides  Previous CABG  Previous PCI  Coronary Atherosclerosis  *Medications* (%)  Anti-platelet agents  Calcium channel blockers  Nitrates  ACE Inhibitors  Betablockers  Statins  Antidiabetics | 20  **103±38**  **136±21**  **75±11**  **212±44**  **50±14**  131±41  127±86  **2**  11  **0.91±0.99**  **74**  **34**  55  **20**  **37**  **49**  17 | 21  **125±71**  **131±26**  **71±13**  **201±53**  **44±12**  122±42  136±104  **8**  13  **1.36±0.93**  **82**  **21**  59  **42**  **56**  **59**  20 | | 0.82  **<0.001**  **0.016**  **0.001**  **0.03**  **<0.001**  0.07  0.33  **0.003**  0.49  **<0.001**  **<0.04**  **0.002**  0.36  **<0.001**  **<0.001**  **0.03**  0.35 | | **29**  **108±38**  **136±20**  **77±10**  **202±45**  **41±14**  **128±51**  152±98  **5**  **11**  **1.42±1.05**  **84**  **38**  **63**  **26**  **41**  **53**  14 | | 27  **113±54**  **130±21**  **75±12**  **191±46**  **38±16**  **117±40**  147±97  **10**  **17**  **1.68±0.95**  **90**  **24**  **69**  **43**  **55**  **57**  15 | | 0.26  **0.038**  **<0.001**  **0.001**  **<0.001**  **0.002**  **<0.001**  0.28  **<0.001**  **<0.001**  **<0.001**  **<0.001**  **<0.001**  **0.004**  **<0.001**  **<0.001**  **0.01**  0.55 | | |  | |  | |  | |  |
|  |  |  | |  | |  | |  | |  | | |  | |  | |  | |  |

Abbreviations as in Table I. Values are reported as mean±SD.
