# Supplementary figures and images for: Long-term prognosis of unheralded myocardial infarction vs chronic angina; role of sex and coronary atherosclerosis burden
Source: BMC Cardiovasc Disord. 2018 Jul 31;18:156. doi: 10.1186/s12872-018-0890-5 (PMC6069774; doi:10.1186/s12872-018-0890-5)

## Slide 1
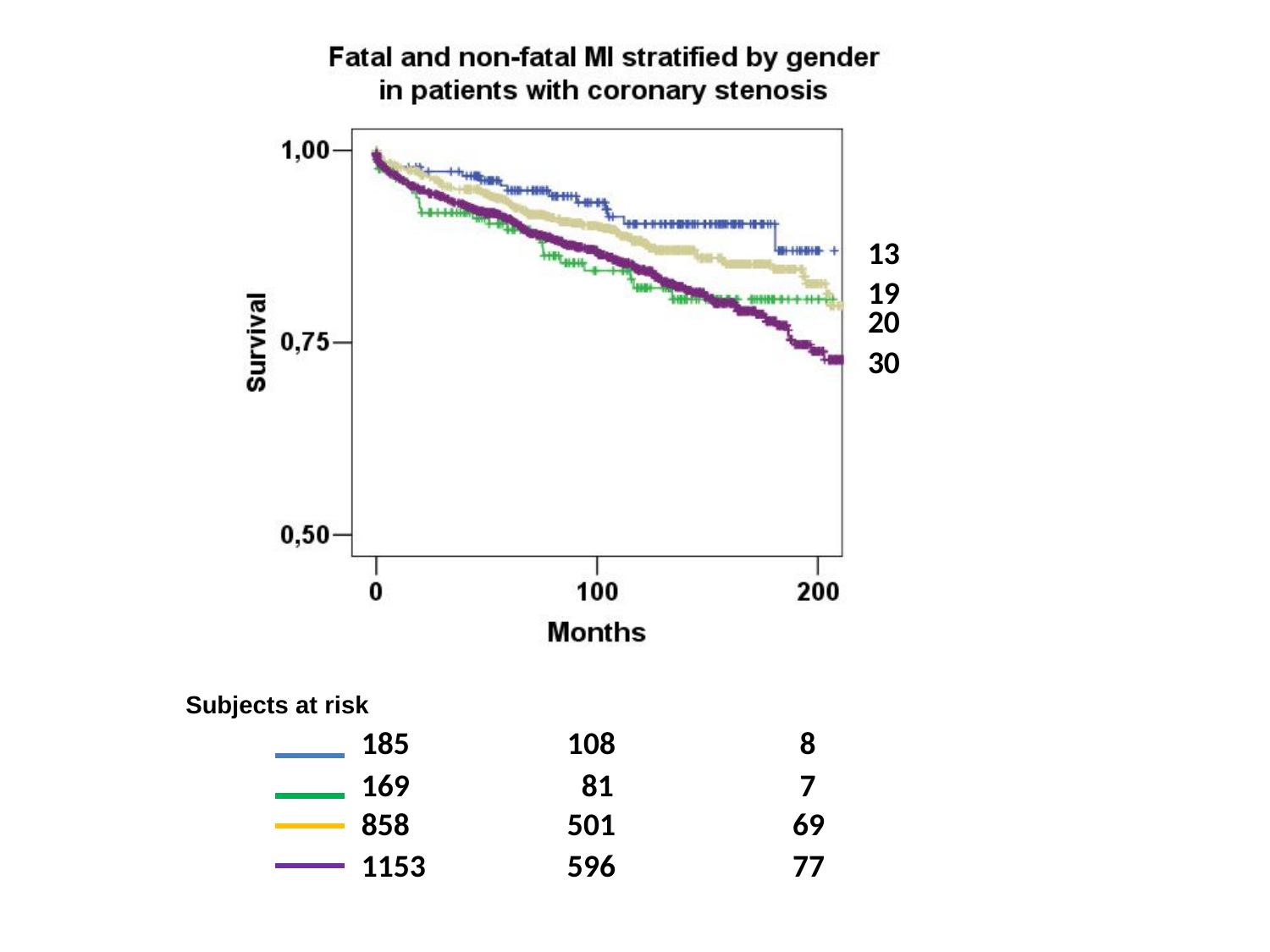

13
19
20
30
 Subjects at risk
	 	185	 108	 8	 	 	169	 81		 7
	 	858	 501	 69
	 	1153	 596	 77

Supplement: Supplementary file 2 — Figure S3a. Kaplan Meyer curves for fatal and non-fatal myocardial infarction in angina pectoris and myocardial infarction excluding patients with normal coronary vessels, stratified by gender. MI = myocardial infarction. Blue line: females in angina group; yellow line: males in angina group; green line: females in myocardial infarction group; purple line: males in myocardial infarction group. (PPTX 118 kb) [file 12872_2018_890_MOESM2_ESM.pptx]

## Slide 1
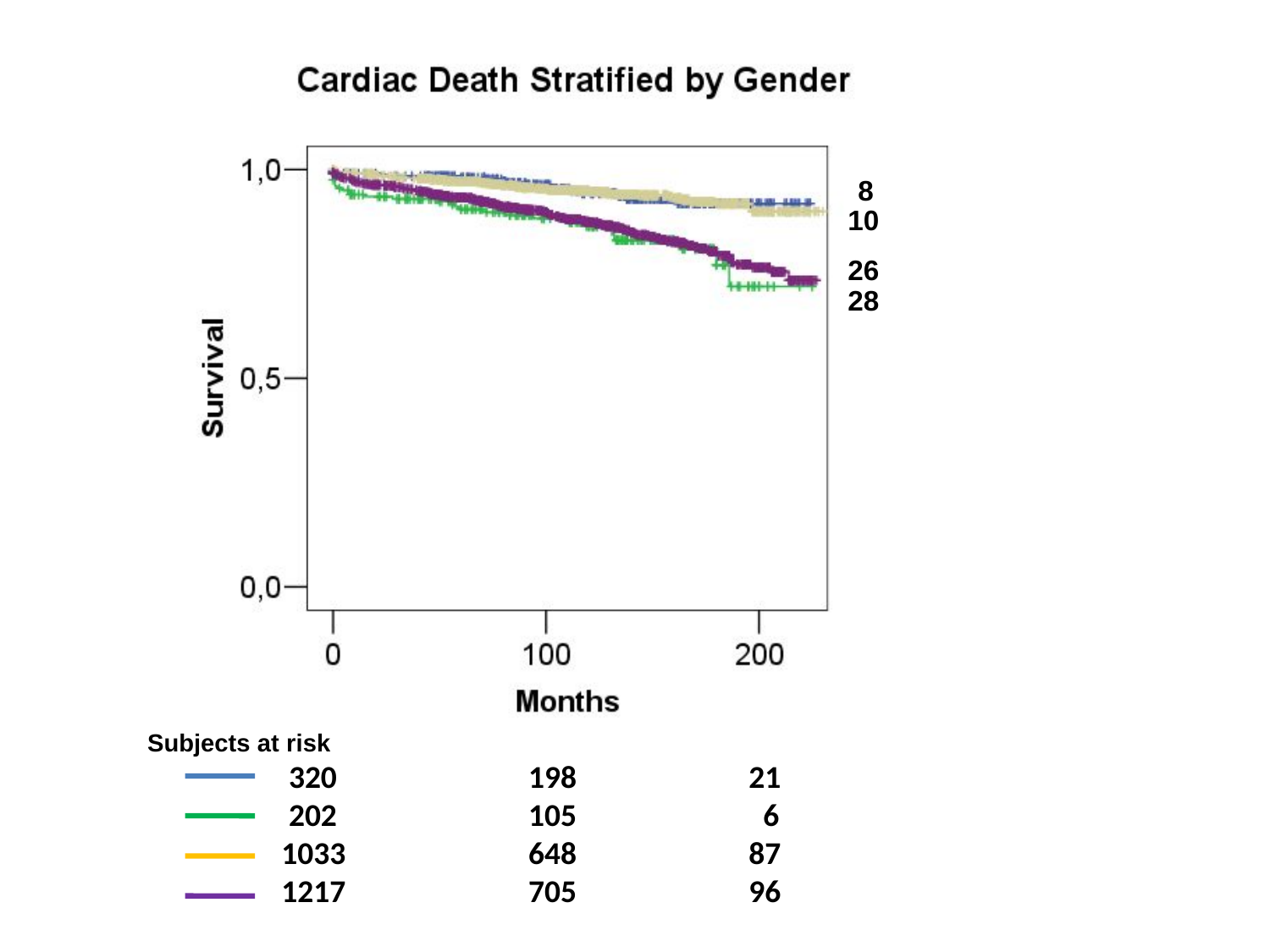

8
10
26
28
Subjects at risk
	 320		198	 21
	 202		105	 6
	 1033		648	 87
	 1217		705	 96

Supplement: Supplementary file 4 — Figure 4. Kaplan Meyer survival curves for cardiac death in angina pectoris and myocardial infarction stratified by gender. Blue line: females in angina group; yellow line: males in angina group; green line: females in myocardial infarction group; purple line: males in myocardial infarction group. (PPT 135 kb) [file 12872_2018_890_MOESM4_ESM.ppt]

## Slide 1
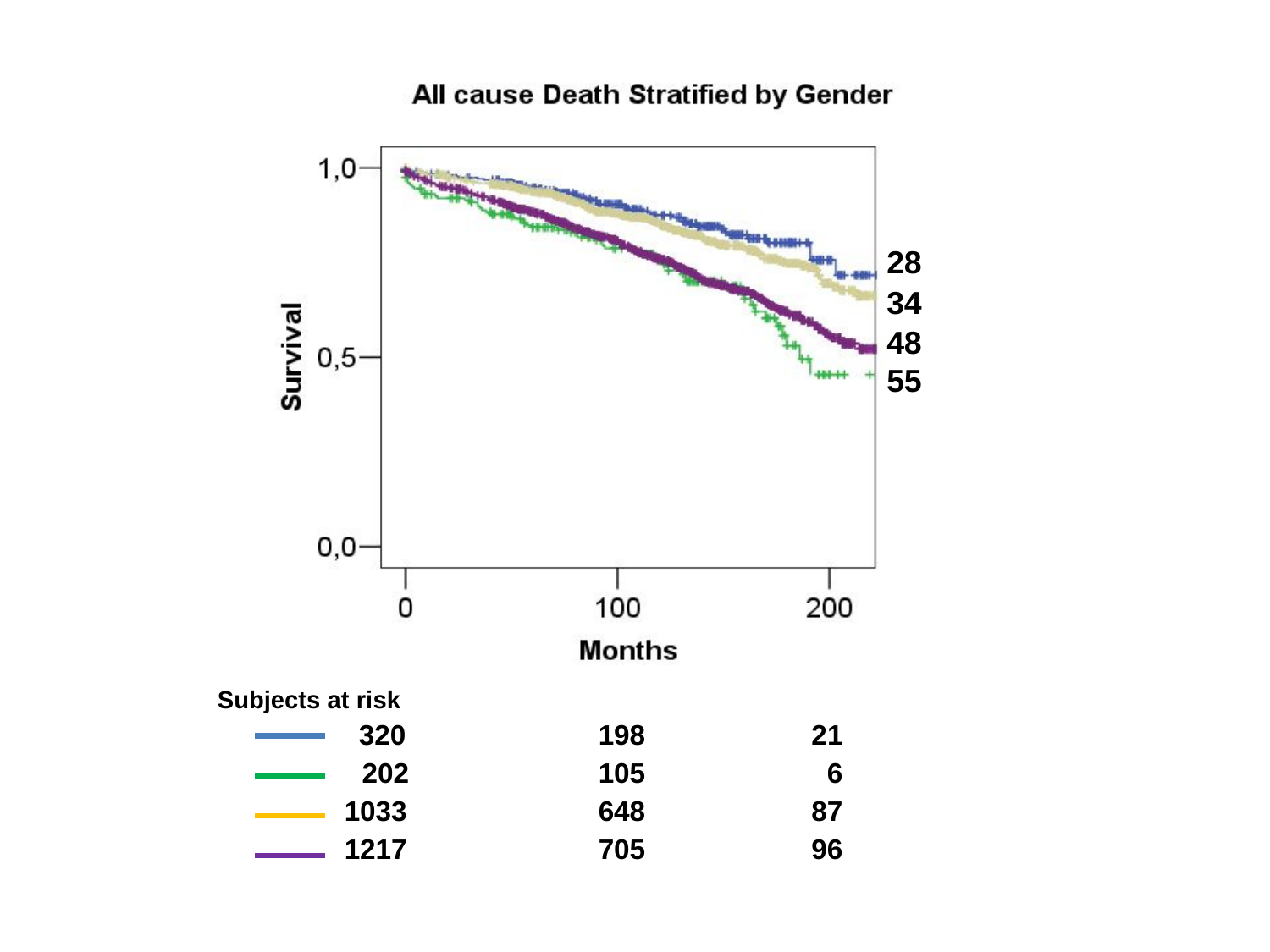

28
34
48
55
Subjects at risk
	 320		198	 21
	 202		105	 6
	1033		648	 87
	1217		705	 96

Supplement: Supplementary file 5 — Figure 5. Kaplan Meyer survival curves for all-cause death in angina pectoris and myocardial infarction stratified by gender. Blue line: females in angina group; yellow line: males in angina group; green line: females in myocardial infarction group; purple line: males in myocardial infarction group. (PPTX 103 kb) [file 12872_2018_890_MOESM5_ESM.pptx]
