# Supplementary material for: Long-term prognosis of unheralded myocardial infarction vs chronic angina; role of sex and coronary atherosclerosis burden
Source: BMC Cardiovasc Disord. 2018 Jul 31;18:156. doi: 10.1186/s12872-018-0890-5 (PMC6069774; doi:10.1186/s12872-018-0890-5)
Supplement: Supplementary file 3 — Table S3. Cox regression estimates of the hazard ratios associated with major follow up events. (DOCX 18 kb) [file 12872_2018_890_MOESM3_ESM.docx]

| **Table S3 .**  Cox regression estimates of the hazard ratios associated with major follow up events |
| --- |

|  | **Hazard Ratio** (95% CI) | **P** |
| --- | --- | --- |
|  | *Non Fatal Myocardial infarction Risk* |  |
| Coronary ATS | 1.33 (1.16-1.51) | **<0.001** |
| Coronary REV | 0.46(0.35-0.61) | **<0.001** |
| MI History | 1.32(1.01-1.72) | **0.04** |
| Sex | 1.58(1.04-2.38) | **0.03** |
| Diabetes | 1.31(0.96-1.80) | 0.09 |
| Smoking | 1.23 (0.93-1.63) | 0.14 |
| Hypertension | 0.89(0.69-1.16) | 0.42 |
| Age | 0.99(0.98-1.01) | 0.85 |
| Family history | 0.80(0.61-1.03) | 0.08 |
| Obesity | 1.07(0.80-1.42) | 0.65 |
| Hypercholesterolemia | 0.97(0.73-1.28) | 0.83 |
|  | *Fatal and Non Fatal*  *Myocardial infarction Risk* |  |
| MI History | 1.55(1.23-1.95) | **<0.001** |
| Diabetes | 1.53(1.19-1.98) | **0.001** |
| Coronary ATS | 1.39 (1.24-1.55) | **<0.001** |
| Coronary REV | 0.48(0.37-0.60) | **<0.001** |
| Sex | 1.37(1.00 -1.92) | **0.05** |
| Family history | 0.77(0.62-0.95) | **0.02** |
| Age | 1.00(0.99-1.02) | 0.40 |
| Smoking | 1.17 (0.92-1.47) | 0.19 |
| Obesity | 1.01(0.79-1.29) | 0.92 |
| Hypertension | 0.97(0.78-1.21) | 0.81 |
| Hypercholesterolemia | 0.86(0.68-1.09) | 0.21 |
|  | *Cardiac Mortality Risk* |  |
| Obesity | 1.26 (0.97-1.63) | 0.07 |
| Coronary ATS | 1.65(1.47-1.87) | **<0.001** |
| Diabetes | 1.74(1.33-2.27) | **<0.001** |
| Age | 1.04(1.02-1.05) | **<0.001** |
| Coronary REV | 0.36(0.28-0.46) | **<0.001** |
| MI history | 2.44(1.84-3.21) | **<0.001** |
| Sex | 1.00(0.71-1.40) | 0.97 |
| Smoking | 0.93(0.72-1.20) | 0.56 |
| Family history | 0.85(0.66-1.08) | 0.18 |
| Hypertension | 0.92(0.72-1.18) | 0.51 |
| Hypercholesterolemia | 0.63(0.48-0.81) | **<0.001** |
|  | *All cause death* |  |
| Diabetes | 1.65(1.38-1.97) | **<0.001** |
| Coronary ATS | 1.37(1.27-1.49) | **<0.001** |
| Age | 1.06(1.04-1.07) | **<0.001** |
| Hypercholesterolemia | 0.64(0.54-0.76) | **0.001** |
| MI history | 1.74(1.47-2.06) | **<0.001** |
| Sex | 1.06(0.85-1.33) | 0.58 |
| Smoking | 1.08(0.91-1.27) | 0.38 |
| Family history | 0.83(0.71-0.97) | **0.02** |
| Hypertension | 1.03(0.88-121) | 0.69 |
| Obesity | 1.11(0.93-1.32) | 0.24 |
| Coronary REV | 0.59(0.49-0.71) | **<0.001** |

ATS=atherosclerosis; MI=myocardial infarction; REV= revascularization.
